# Antiaging Effects of *Vicatia thibetica de Boiss* Root Extract on *Caenorhabditis elegans* and Doxorubicin-Induced Premature Aging in Adult Mice

**DOI:** 10.1155/2021/9942090

**Published:** 2021-08-06

**Authors:** Wenwen Liu, Yunhui Guan, Sicong Qiao, Jiqun Wang, Keting Bao, Zhifan Mao, Liang Liao, Alexey Moskalev, Bei Jiang, Jin Zhu, Conglong Xia, Jian Li, Zelan Hu

**Affiliations:** ^1^State Key Laboratory of Bioreactor Engineering, Shanghai Key Laboratory of New Drug Design, East China University of Science and Technology, 130 Mei Long Road, Shanghai 200237, China; ^2^College of Pharmacy and Chemistry, Dali University, 5 Xue Ren Road, Dali, Yunnan 671000, China; ^3^. Laboratory of Geroprotective and Radioprotective Technologies, Institute of Biology, Komi Science Centre, Ural Branch, Russian Academy of Sciences, 28 Kommunisticheskaya st, Syktyvkar 167982, Russia; ^4^Institute of Materia Medica, Dali University, 5 Xue Ren Road, Dali, Yunnan 671000, China; ^5^Clinical Medicine Scientific and Technical Innovation Center, Shanghai Tenth People's Hospital, Tongji University School of Medicine, Shanghai 200092, China

## Abstract

The roots of *Vicatia thibetica de Boiss* are a kind of Chinese herb with homology of medicine and food. This is the first report showing the property of the extract of *Vicatia thibetica de Boiss* roots (HLB01) to extend the lifespan as well as promote the healthy parameters in *Caenorhabditis elegans* (*C. elegans*). For doxorubicin- (Doxo-) induced premature aging in adult mice, HLB01 counteracted the senescence-associated biomarkers, including P21 and *γ*H2AX. Interestingly, HLB01 promoted the expression of collagen in *C. elegans* and mammalian cell systemically, which might be one of the essential factors to exert the antiaging effects. In addition, HLB01 was also found as a scavenger of free radicals, thereby performing the antioxidant ability. Lifespan extension by HLB01 was also dependent on DAF-16 and HSF-1 via oxidative stress resistance and heat stress resistance. Taken together, overall data suggested that HLB01 could extend the lifespan and healthspan of *C. elegans* and resist Doxo-induced senescence in mice via promoting the expression of collagen, antioxidant potential, and stress resistance.

## 1. Introduction

Aging is a process which includes loss of physiological integrity, impaired functions, and increased vulnerability to death. The well-known hallmarks of aging include telomere attrition, loss of proteostasis, mitochondrial dysfunction, cellular senescence, and stem cell exhaustion [1]. In recent years, some antiaging interventions like SA-*β*-gal-activated prodrugs and senolytics have appeared [2–4]. Several senescence biomarkers such as SA-*β*-gal, P16^INK4A^, P53, P21, and *γ*H2AX have been widely used to evaluate the effects of antiaging interventions [5, 6]. Damage accumulation of extracellular matrix (ECM) proteins is called a missing hallmark of aging, which has recently drawn an increased attention [7]. Age-related changes of the components of the ECM (collagen, elastin, MMPs, cathepsins, etc.) could disrupt the homeostasis of tissue microenvironment and facilitate the age-related pathologies [8, 9]. Therefore, the deterioration in ECM integrity has been implicated in many age-dependent diseases, such as diabetes, cancer, chronic liver diseases, and cardiovascular diseases [9–11]. Interestingly, it was shown that the cells in a proper microenvironment could live significantly longer than those in the original environment [12, 13], which also demonstrated the importance of ECM maintenance.

Collagens are the most abundant ECM proteins in the organism and major structural proteins of the ECM [14]. The structural changes and degradation of collagen are associated with bone and cartilage deterioration, skin wrinkling, and cardiovascular and respiratory malfunctions [15]. Moreover, it has been shown in the studies that the increased collagen expression is a shared feature of multiple conserved longevity pathways including *Caenorhabditis elegans* (*C. elegans*) [11]. Promoting the biosynthesis of collagens during the lifetime is required and beneficial for the healthy aging and longevity in *C. elegans* [11]. Furthermore, deficits in matrix metalloproteinases 14 (MMP14), which are involved in collagen degradation and ECM remodeling, lead to premature aging, short lifespan, and aging in mice [16]. Taken together, all these findings suggest that strategies for promoting the collagen and ECM function systemically may be widely applicable in delaying aging. However, interventions which promote collagen and ECM function are rarely reported.

**Figure 1:**
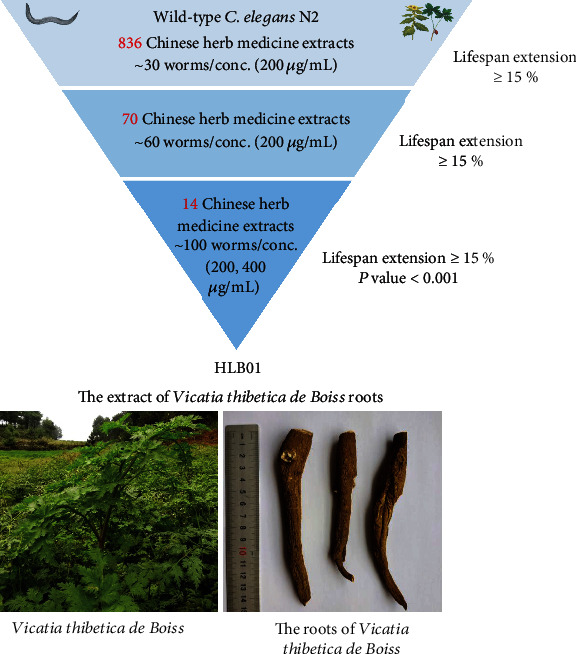
A Chinese herbal medicine library-based phenotypic screening led to the discovery of HLB01.

Because of fast reproduction, short life cycle, and conserved sequence with human genes, *C. elegans* was widely used in antiaging drug candidate screening and novel target finding [17, 18]. Moreover, many compounds, which were discovered to have antiaging effects by the lifespan screening on *C. elegans*, also showed similar effects in the mouse model [19–21]. In our previous work, we screened our in-house 1,386 marketable drugs by testing their effect on the lifespan of *C. elegans* and antihypertensive drug verapamil was found to have an antiaging effect [22]. Herein, in this study, we screened 836 extracts of Chinese herbal medicine which have been used for a long time in humans. After several rounds of lifespan tests, the extract of *Vicatia thibetica de Boiss* roots (HLB01) was selected for further study based on its activity (Figure 1). The root of *Vicatia thibetica de Boiss* is a kind of herb with homology of medicine and food, suggesting that HLB01 has a great potential to be developed for its antiaging potential with high safety.

The common chemotherapeutic drug doxorubicin (Doxo) has a proven effect of inducing senescence and liver toxicity in rodents and humans [23]. In our study, Doxo-induced premature aging mice were selected as the animal models for evaluating the antiaging pharmacological effect of HLB01 via biomarkers of aspartate aminotransferase (AST), alanine aminotransferase (ALT), P21, and *γ*H2AX. Then, the mechanisms of HLB01 were studied with the emphasis on collagen expression, antioxidative ability, and stress resistance.

## 2. Materials and Methods

### 2.1. Preparation of HLB01 Extract

10 kg of dried roots of *Vicatia thibetica de Boiss* was crushed with a shredder, decocted with distilled water for 3 times at 80°C with a solid-liquid ratio of 1 : 10, 90 min each time. Thereafter, extracts were combined, decompressed, and concentrated. This protocol resulted in 4.55 kg extract (HLB01), and the calculated yield was 45.5%.

### 2.2. *C. elegans* Maintenance and Strains

Strains were cultured on nematode growth medium (NGM) agar plates at 20°C. *C. elegans* strains used in this study included the following (name, genotype, and origin): N2, *C. elegans* wild isolate, CGC; CF1038, *daf-16*(mu86) I, CGC; PS3551, *hsf-1*(sy441) I, CGC.

### 2.3. Lifespan Analysis

Live *Escherichia coli* strain OP50 was used as the food source. Worms were synchronized and transferred to NGM agar plates at L4 stage. We set control and HLB01-treated groups, with approximately 100 worms per group. To avoid progeny hatching, 50 *μ*g/mL of 5-fluorodeoxyuridine (FudR) was added to the agar plates from day 0 to day 10. Worms were treated with HLB01 (200 *μ*g/mL or 400 *μ*g/mL) only for the first 10 days. Worms were counted every day and transferred to the fresh plates every 3 days until all the worms were dead. Worms which exhibited exploded vulva phenotype and crawled off the plate were censored. Three replicate experiments were conducted. The log-rank (Mantel-Cox) test was used to calculate the *P* values.

### 2.4. Thrashing Assay

Thrashing assay was conducted as described previously [24]. Wild-type worms were cultured on NGM plates as per the lifespan assay protocol. 20-30 worms were prepared for control and HLB01 (200 *μ*g/mL) treatment groups, respectively. One worm was removed in an M9 buffer drop on an NGM plate without OP50 bacteria and allowed to adapt for 30 s. Then, we counted the number of thrashes for 30 seconds. Any change in the midbody bending direction was referred to as a thrash [25]. Thrashes were counted on days 3, 8, and 13. Three replicate experiments were conducted. A two-way ANOVA along with the Sidak multiple comparisons test was used to calculate *P* values, and error bars represented SEM.

### 2.5. Pharyngeal Pumping Assay

Pharyngeal pumping assay was conducted as per previously reported protocol [26]. Wild-type worms were cultured on NGM according to the lifespan assay protocol. 20-30 worms were prepared for the pharyngeal pumping assay for control and HLB01 (200 *μ*g/mL) treatment groups. On days 1, 3, 5, 7, 9, and 11, the pharyngeal pumping rate was tested by quantifying the contractions of the pharynx over a period of 30 seconds. Three replicate experiments were conducted. A two-way ANOVA along with the Sidak multiple comparisons test was used to calculate *P* values, and error bars represented SEM.

### 2.6. Bacterial Growth Assay

Bacterial growth assay was conducted as described previously [27]. Initially, a single colony of OP50 was inoculated in LB media and cultured at 37°C. For each group, 30 *μ*L of bacterial culture (OD_600_ = 0.4) was dropped to an NGM plate and cultured at 20°C. Bacteria were washed off using 1 mL M9 buffer, and OD_600_ was measured every 12 h, with M9 buffer as the blank control. OD was assessed using a Hitachi U-2910 spectrometer with a 10 mm quartz cuvette. Three replicate experiments were conducted. An unpaired *t*-test was used to calculate the *P* values, and error bars represented SEM.

### 2.7. Mouse Experiments

**Figure 2:**
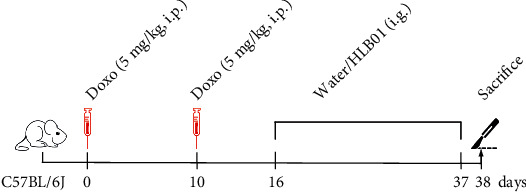
Experimental design for Doxo-induced premature aging and HLB01 treatment of those aged mice. Doxo: two times i.p. at 5 mg/kg. HLB01: from day 16 to 37, i.g. at 1.2 g/kg and 2.4 g/kg.

Male C57BL/6J mice were purchased from Beijing Vital River Laboratory Animal Technology Co., Ltd. (Beijing, China). Mouse experiments were conducted at Shanghai SLAC Laboratory Animal Co., Ltd. (Shanghai, China), permit number: SYXK (Shanghai) 2017-0008, contract no: SLAC2020072806. Animal procedures were carried out according to the National Institutes of Health (NIH) guidelines. Mouse experiments were conducted as per the previously described protocol [28]. We have tried three doses of Doxo (2 mg/kg, 5 mg/kg, and 10 mg/kg) to induce premature aging in adult mice. Among them, the dose of 5 mg/kg we selected showed both a high survival rate and significant aging indexes. C57BL/6J mice (10 weeks, male) were employed to evaluate the efficacy of HLB01 in vivo. In the blank group (*n* = 10), mice were treated with saline. In the Doxo group (*n* = 15), mice were intraperitoneally injected with 5 mg/kg Doxo twice on day 0 and day 10 to induce aging. In Doxo-HLB01 groups (*n* = 15, each group), the Doxo-treated mice were administered intragastrically with HLB01 (two groups: one received HLB01: 1.2 g/kg and another group received HLB01: 2.4 g/kg) by intragastric route from day 16 to day 37 (Figure 2). The doses of HLB01 were set by referring to the dose of *Seselopsis* extract in an antifatigue effect [29]. Mice were administrated with the same volume of extracts in 1.2 g/kg and 2.4 g/kg groups. The mortality rate of mice after Doxo and HLB01 treatment was recorded. Thereafter, mice were sacrificed on day 38.

### 2.8. Test of AST and ALT Level

Plasma sample of treated and untreated mice was collected and spun for 20 minutes at 3000 rpm. The supernatant was collected and sent to KingMed Diagnostics, Shanghai, China, for AST and ALT measurement. The level of AST was tested according to the protocol of aspartate aminotransferase (ASTL) (Roche). A 9 *μ*L supernatant was incubated with 40 *μ*L R1 and 17 *μ*L R2 and placed at 37°C for 10 min. Then, the absorbance of NADH (*λ* = 340 nm) was tested. AST could catalyze the transfer of amino groups between L-aspartate and 2-ketoglutaric acid and then generate L-glutamic acid and oxobutanedioic acid. Under the catalysis of malate dehydrogenase (MDH), oxaloacetic acid reacts with reduced nicotinamide adenine dinucleotide NADH to form NAD^+^. The oxidation rate of NADH is proportional to the catalytic activity of AST. The level of ALT was tested according to the protocol of alanine aminotransferase acc. to IFCC (ALTL) (Roche). A 9 *μ*L supernatant was incubated with 59 *μ*L R1 and 17 *μ*L R2 and placed at 37°C for 10 min. Then, the absorbance of NADH (*λ* = 340 nm) was tested. L-Alanine and 2-ketoglutaric acid were catalyzed by ALT to form pyruvic acid. Under the catalysis of lactate dehydrogenase, pyruvic acid and NADH produce L-lactic acid and NAD^+^. The oxidation rate of NADH was proportional to the activity of ALT. Therefore, levels of AST or ALT could be calculated by absorbance of NADH (*λ* = 340 nm). An unpaired *t*-test was used to calculate the *P* values, and error bars represented SEM.

### 2.9. Quantitative Real-Time PCR

The samples of wild-type worms, liver, and kidney were prepared, and their RNA was extracted from them using the Total RNA Kit (OMEGA, BioTek). Then, reverse transcription was conducted using Hifair® 1st Strand cDNA Synthesis SuperMix for qPCR (gDNA digester plus) (Yeasen, 11123ES10) by LongGene A200. Hieff UNICON® Universal Blue qPCR SYBR Green Master Mix (Yeasen, 11184ES03) was used to perform qPCR (BIO-RAD, CFX96). Three replicate experiments were conducted. An unpaired *t*-test was used to calculate the *P* values, and error bars represented SEM.

### 2.10. Western Blot

Protein was extracted from cells, liver, and kidney using RIPA lysis buffer (Yeasen). Protein extracts were quantified by the BCA Protein Quantification Kit (Yeasen). For *γ*H2AX, 12% sodium dodecyl sulfate-polyacrylamide gel electrophoresis (SDS-PAGE) was conducted to separate the protein bands, and then, proteins were transferred from gel to the PVDF membrane, blocked with 5% milk powder in TBST for 1 hour. Membranes were incubated with specific antibodies for *γ*H2AX (ab81299, Abcam) and tubulin (ab6160, Abcam). For collagen, 8% SDS-PAGE was run. Membranes were incubated with specific antibodies for GAPDH (60004-1-lg, Proteintech) and Collagen I (ab260043, Abcam). Then, immunocomplexes were detected by Peroxidase-Conjugated Goat Anti-Rabbit IgG (H+L) (33101ES60, Yeasen), Peroxidase-Conjugated Goat Anti-Rat IgG (H+L) (33301ES60), and Peroxidase AffiniPure Goat Anti-Mouse IgG (H+L) (33201ES60, Yeasen) followed by enhanced chemoluminescence (New Cell & Molecular Biotech). The membrane was finally photographed by imaging techniques (Tanon-4600SF). ImageJ was used to quantify the intensity.

### 2.11. RNA-seq

Wild-type worms of control and HLB01 (200 *μ*g/mL) groups were cultured according to the lifespan assay. At day 10, worms were collected for RNA-seq in Shanghai Majorbio Bio-pharm Technology Co., Ltd. Data was analyzed using the free online platform of Majorbio Cloud Platform. The accession number for RNA-seq reported in this paper is GEO: GSE168322.

### 2.12. Collagen Assay

Collagen assay was conducted as described previously [30]. Wild-type worms from the control and HLB01 (200 *μ*g/mL) groups were cultured according to the lifespan assay. At day 10, worms were collected and washed by M9 for three times. Thereafter, collagen levels were measured by detecting hydroxyproline using the Hydroxyproline (HYP) Content Assay Kit (Solarbio, BC0250) according to the manufacturer's instructions. Three replicate experiments were conducted. An unpaired *t*-test was used to calculate the *P* values, and error bars represented SEM.

### 2.13. Cell Viability Assay

Human embryonic lung fibroblast MRC-5 cells were cultured in MEM (Gibco) supplemented with 10% fetal bovine serum (Gibco), 1% sodium pyruvate (BI), 1% NEAA (BI), and 1% penicillin-streptomycin (Yeasen). Human skin fibroblast CCC-ESF-1 were cultured in DMEM (Gibco) supplemented with 10% fetal bovine serum (Gibco) and 1% penicillin-streptomycin (Yeasen). Cells were maintained in an incubator at 37°C under 5% CO_2_. For testing cell viability, cells were seeded into a 96-well plate at 1 × 10^4^ cells per well and treated with varying concentrations of HLB01 (6.25, 12.50, 25.00, 50.00, 100.00, and 200 *μ*g/mL) for 48 h. After that, CCK8 solution was added to the wells and incubated at 37°C for 2 h. Thereafter, absorbance was measured at 450 nm using a microplate reader (BioTek Instruments, Synergy, H1). Three replicate experiments were conducted. An unpaired *t*-test was used to calculate the *P* values, and error bars represented SEM.

### 2.14. 2,2-Diphenyl-1-Picrylhydrazyl (DPPH) Radical Scavenging Assay

DPPH radical scavenging assay was conducted as described previously [31]. 100 *μ*L HLB01 of different concentrations were incubated with 100 *μ*L of 200 *μ*M DPPH solution in a 96-well plate. The plate was incubated at room temperature for 30 min, away from any source of light. Then, absorbance was measured at 517 nm. A blank without DPPH addition was prepared and measured for each sample in a similar way. DPPH radical scavenging ability (%) = [(*A*_0_ − *A*_1_)/*A*_0_]∗100. *A*_0_ and *A*_1_ represent the absorbance of the control and the sample, respectively. Three replicate experiments were conducted.

### 2.15. Measurement of Reactive Oxygen Species (ROS) Level

2′,7′-dichlorodihydrofluorescein diacetate (DCFH-DA) (Yeasen) was used to detect the ROS level. DCFH-DA is a ROS sensor that gets deacetylated by intracellular esterases to release a nonfluorescent compound (DCFH), which is later oxidized by ROS into 2′,7′-dichlorofluorescein (DCF) emitting green fluorescence that could be detected by fluorescence microscope to indicate the intracellular ROS levels [32]. Worms were cultured according to the lifespan assay. For the whole worms, ROS level determination was conducted by the previously described method [33]. About 30~40 worms of the HLB01 (200 *μ*g/mL) group and control group were, respectively, collected at day 6 and washed three times by M9 buffer. After that, the worms were incubated with 50 *μ*M DCFH-DA in the M9 for 30 min. Fluorescence was measured after that by an inverted fluorescence microscope (Nikon Eclipse Ti2 microscope) at 100× magnification. ImageJ was used to quantify intensity. Three replicate experiments were conducted. An unpaired *t*-test was used to calculate the *P* values, and error bars represented SEM. For worm lysis solution, ROS levels were detected as described previously [34]. About 500 worms of the HLB01 (200 *μ*g/mL) group and control group were, respectively, collected at day 6, and washed by M9 buffer for three times to eliminate the bacterial contamination. After that, worms were washed by PBS once in a 1.5 mL tube. These worms were frozen in liquid nitrogen immediately and then thawed immediately at room temperature. Thereafter, sonication (UXI, JY92-IIN) was used to further lyse these worms. Lysates were centrifuged (12,000 rpm at 4°C for 15 min) to obtain the supernatants. Supernatants were quantified by the BCA Protein Quantification Kit (Yeasen). Next, a supernatant containing 50 *μ*g protein and 200 *μ*L of 250 *μ*M DCFH-DA in PBS were incubated together and scanned using a fluorospectro photometer (Techcomp, F-4700) immediately. The ROS level was determined by the fluorescence intensity. Three replicate experiments were conducted.

### 2.16. Statistical Analysis

All quantitative data are presented as the means ± SEM. Statistical analyses included unpaired *t*-test, log-rank (Mantel-Cox) test, and two-way ANOVA along with the Sidak multiple comparisons test. All figures were generated using GraphPad Prism 6 and Microsoft Office.

## 3. Results

### 3.1. HLB01 Extends Lifespan and Healthspan of *C. elegans*

**Figure 3:**
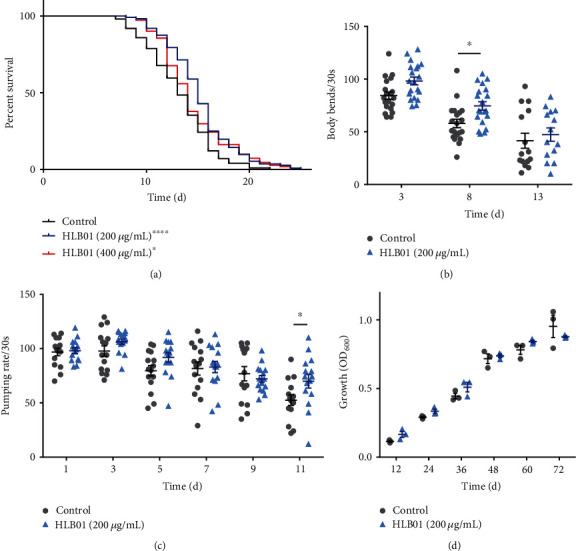
HLB01 extends lifespan and healthspan of *C. elegans*. (a) HLB01 increased the lifespan of *C. elegans* (wild-type) at 200 *μ*g/mL and 400 *μ*g/mL. (b) HLB01 promoted the ability of body bends of wild-type worms, significantly on day 8. (c) HLB01 increased the pharyngeal pumping rate of wild-type worms significantly on day 11. (d) HLB01 did not reduce bacterial growth. The log-rank (Mantel-Cox) test was used to calculate the *P* values in (a). A two-way ANOVA along with Sidak multiple comparisons test was used to calculate *P* values, and error bars represented SEM in (b) and (c). Multiple *t*-tests were used to calculate the *P* values, and error bars represented SEM in (d). ^∗^*P* < 0.05; ^∗∗∗∗^*P* < 0.0001.

To examine the antiaging effects of HLB01, we first analyzed the lifespan extension of *C. elegans* after treatment with HLB01. Compared to the control group, treatment with 200 *μ*g/mL HLB01 led to a lifespan extension by 14.79% (^∗^*P* < 0.05) in *C. elegans* and that with 400 *μ*g/mL HLB01 by 9.61% (^∗∗∗∗^*P* < 0.0001) (Figure 3(a), Table S1). Based on the above, we chose the concentration of 200 *μ*g/mL for further studies. We further monitored the age-dependent changes of aging biomarkers to assess the physiological status of aging in *C. elegans*. The effect of HLB01 on the motility of *C. elegans* was evaluated by counting the body bends times every 30 seconds on day 3, day 8, and day 13 of adulthood. HLB01-treated groups showed a more intense swinging motion, especially on day 8 (^∗^*P* < 0.05) (Figure 3(b)). Pharyngeal pumping assay of *C. elegans* also showed that HLB01 treatment did not have any adverse effect on the pumping rate (on days 1, 3, 5, 7, 9, and 11) and promoted healthy parameter on day 11 significantly (^∗^*P* < 0.05) (Figure 3(c)). The above results showed that HLB01 not only extended the lifespan but also promoted healthspan and physiological parameters in *C. elegans*. Furthermore, the cell viability test in human fetal lung fibroblasts cells (MRC-5) was investigated, and the results demonstrated no cytotoxicity of HLB01 even at a high concentration of 200 *μ*g/mL (Figure S1).

Next, to evaluate whether HLB01 increased the survival of *C. elegans* by reducing the bacterial growth, we examined the growth of bacteria (*Escherichia coli*, OP50) treated with HLB01. Bacterial growth was found not to be inhibited by HLB01, which suggested the lifespan extension of HLB01 was not due to the inadequate food provision (Figure 3(d)).

### 3.2. HLB01 Counteracts Doxorubicin-Induced Aging in Mice

**Figure 4:**
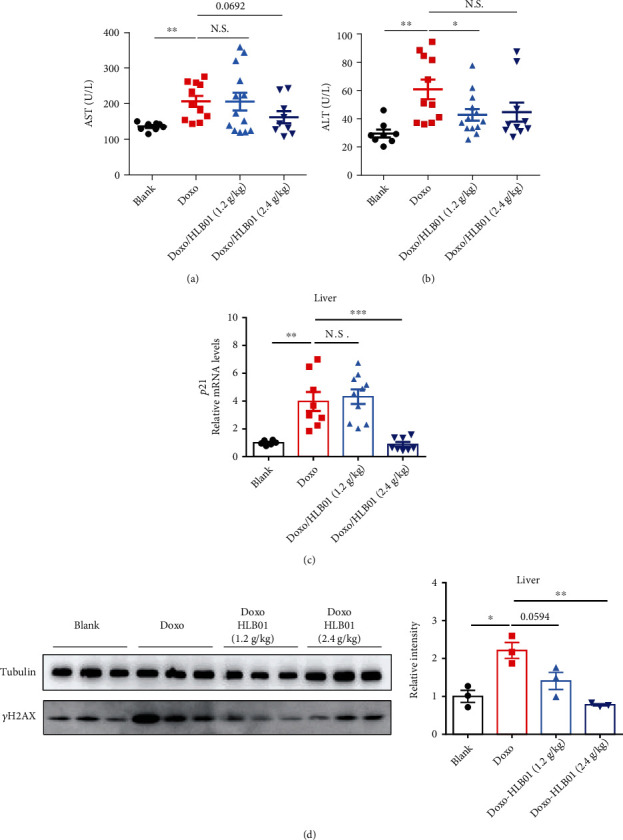
HLB01 counteracts Doxo-induced premature aging in adult mice. (a) HLB01 intended to counteract the Doxo-induced elevation of plasma AST levels. (b) HLB01 (1.2 g/kg) significantly counteracted Doxo-induced elevation of plasma ALT levels. (c) HLB01 (2.4 g/kg) decreased the Doxo-induced increase of *p21* mRNA level. (d) HLB01 (2.4 g/kg) downregulated Doxo-induced increase of *γ*H2AX expression. An unpaired *t*-test was used to calculate the *P* values, and error bars represented SEM in (a–d). ^∗^*P* < 0.05; ^∗∗^*P* < 0.01; ^∗∗∗^*P* < 0.001; N.S.: not significant.

We further studied the antiaging effect of HLB01 in mammals. The mortality rate of mice in the Doxo group (*n* = 15) and Doxo/HLB01 (2.4 g/kg) group (*n* = 15) was 13.3%. There were no mice that died in the blank group (*n* = 15) and Doxo/HLB01 (1.2 g/kg) group (*n* = 15). As a drug with strong chemical toxicity, Doxo increased the plasma levels of AST and ALT, which are known biomarkers for liver damage. HLB01 counteracted the Doxo-induced elevation of plasma AST (1.2 g/kg, N.S.; 2.4 g/kg, *P* = 0.0692) and ALT (1.2 g/kg, ^∗^*P* < 0.05; 2.4 g/kg, N.S.) (Figures 4(a) and 4(b)). Moreover, aging-related P21 and *γ*H2AX were detected. Compared with the Doxo group, HLB01 decreased this transcriptional upregulation of *p21* in the liver remarkably (1.2 g/kg, N.S.; 2.4 g/kg, ^∗∗∗^*P* < 0.001) (Figure 4(c)). Excitingly, HLB01 downregulated the protein levels of *γ*H2AX in the liver (1.2 g/kg, *P* = 0.0594; 2.4 g/kg, ^∗∗^*P* < 0.01) (Figure 4(d)). Together, these results demonstrated that HLB01 is effective in reducing Doxo-induced aging along with the upregulation of AST, ALT, *p21* mRNA, and *γ*H2AX protein levels.

### 3.3. HLB01 Promotes Collagen Expression in *C. elegans* and Mammalian Cell

**Figure 5:**
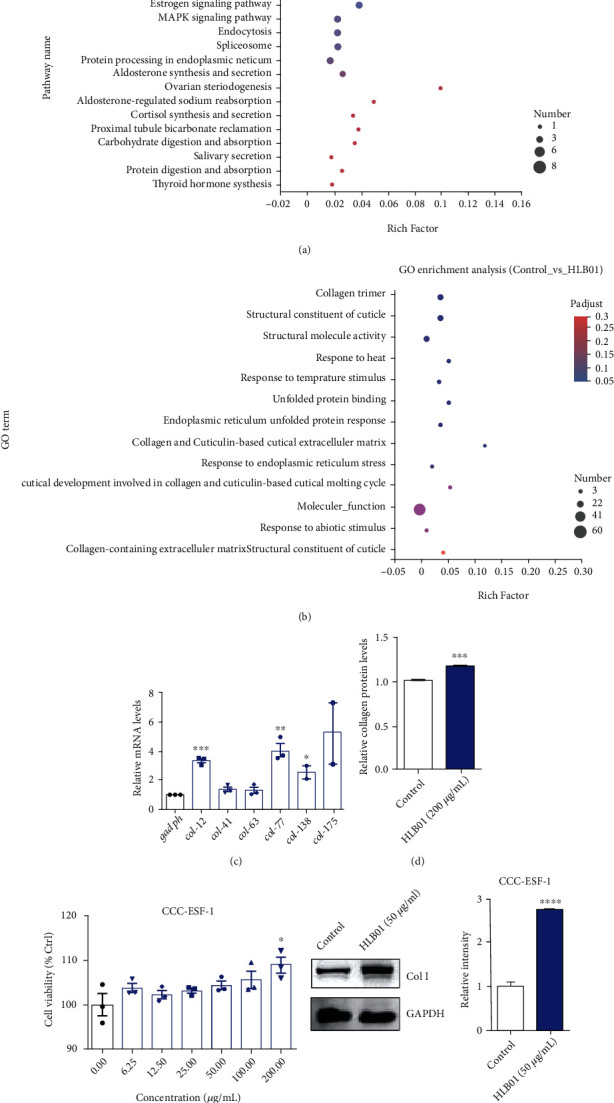
HLB01 promotes collagen expression in *C. elegans* and a mammalian cell. (a) KEGG analysis between the control and HLB01-treated groups in *C. elegans* revealed that HLB01 mainly affects the longevity-regulating pathway. (b) GO enrich analysis between the control and HLB01-treated groups in *C. elegans* revealed that HLB01 mainly affected the collagen-based extracellular matrix. (c) HLB01 upregulated the expression of collagen-related genes (*col-12*, *col-41*, *col-63*, *col-77*, *col-138*, and *col-175*) in *C. elegans*. (d) HLB01 (200 *μ*g/mL) increased the expression of collagen in *C. elegans* by detecting the level of hydroxyproline. (e) The cell viability of HLB01 in human skin fibroblast (CCC-ESF-1). (f) HLB01 (50 *μ*g/mL) upregulates the expression of collagen type I in human skin fibroblast. An unpaired *t*-test was used to calculate the *P* values, and error bars represented SEM in (c–f). ^∗^*P* < 0.05; ^∗∗^*P* < 0.01; ^∗∗∗^*P* < 0.001; ^∗∗∗∗^*P* < 0.0001.

To decipher the mechanism of HLB01-mediated antiaging effect, we used RNA-seq to analyze which process was altered by HLB01 in *C. elegans*. Kyoto Encyclopedia of Genes and Genomes (KEGG) analysis showed that the longevity-regulating pathway was dramatically changed (Figure 5(a)). Gene Ontology (GO) enrichment analysis and classification showed that HLB01 mainly affected the collagen-based extracellular matrix (Figure 5(b)). We further examined transcriptional level changes of collagen genes via quantitative real-time polymerase chain reaction (qRT-PCR) analysis and found that mRNA expression of *col-12*, *col-77*, and *col-138* was significantly increased in HLB01-treated worms (Figure 5(c)). Moreover, we also observed the increased level of collagen in HLB01-treated worms as detected by the level of hydroxyproline, compared to the control group (^∗∗∗^*P* < 0.001) (Figure 5(d)).

In order to explore the collagen level in mammalian cells, we chose a strain of human skin fibroblast (CCC-ESF-1) for evaluating the collagen-promoting ability of HLB01. In the first place, we conducted a cell viability assay to evaluate appropriate concentrations of HLB01. HLB01 showed no toxicity to human skin fibroblast and had no influence in cell proliferation of human skin fibroblast except at the concentration of 200 *μ*g/mL (Figure 5(e)). In order to avoid the influence of cell proliferation to collagen level, we tested collagen protein levels in human skin fibroblasts at concentration of 50 *μ*g/mL but not 200 *μ*g/mL in human skin fibroblasts. As a result, western blotting of collagen also revealed that the protein levels of collagen (type I) are much higher in the HLB01-treated group than in the control group (^∗∗∗∗^*P* < 0.0001) (Figure 5(f)). In summary, we found an antiaging extract which promoted collagen protein levels both *in vitro* and *in vivo*. These results suggested that the antiaging effect of HLB01 is related to the upregulation of collagen.

### 3.4. HLB01 Decreases Oxidative Stress in *C. elegans*

**Figure 6:**
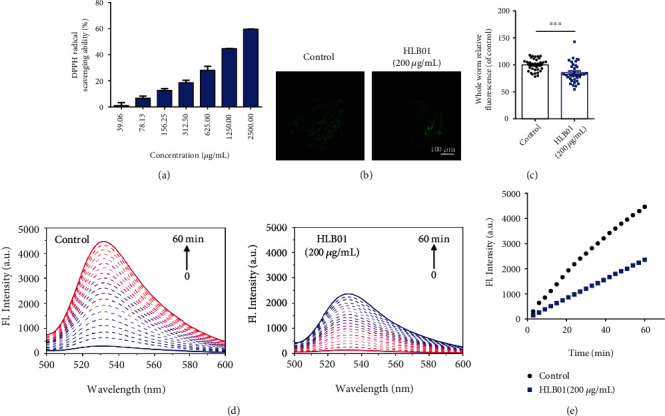
HLB01 decreases oxidative stress in *C. elegans*. (a) HLB01 showed concentration-dependent DPPH radical scavenging ability. (b, c) HLB01 decreased the ROS levels in worms detected by DCFH-DA. (c) is the statistical analysis of (b). (d) Fluorescence intensity of DCFH-DA incubated with worm lysis solution in one hour. (e) HLB01 decreased ROS levels in worm lysis solution, compared with the control group. An unpaired *t*-test was used to calculate the *P* values, and error bars represented SEM in (c). ^∗∗∗^*P* < 0.001.

Next, we tested the radical-scavenging effect of HLB01 to evaluate its antioxidant property *in vitro*. HLB01 showed concentration-dependent DPPH radical scavenging ability (Figure 6(a)). Next, to examine whether the antioxidative capacity is maintained *in vivo*, we tested the ROS level via the DCFH-DA assay in *C. elegans*. Compared to the control group, the ROS level in whole worms of the HLB01-treated group was significantly decreased on day 6 (^∗∗∗^*P* < 0.001) (Figures 6(b) and 6(c)). The ROS level was detected by another protocol also wherein worm lysis solution of different groups was collected, respectively, incubated with DCFH-DA, and detected by a fluorospectro photometer. It was observed that the ROS levels gradually increased with the time, but the level of the HLB01-treated group was lower than the control group (Figures 6(d) and 6(e)). Therefore, it could be inferred that HLB01 decreased the oxidative stress and showed antioxidative ability in *C. elegans*.

### 3.5. HLB01 Enhances Stress Resistance of *C. elegans* via HSF-1 and DAF-16

**Figure 7:**
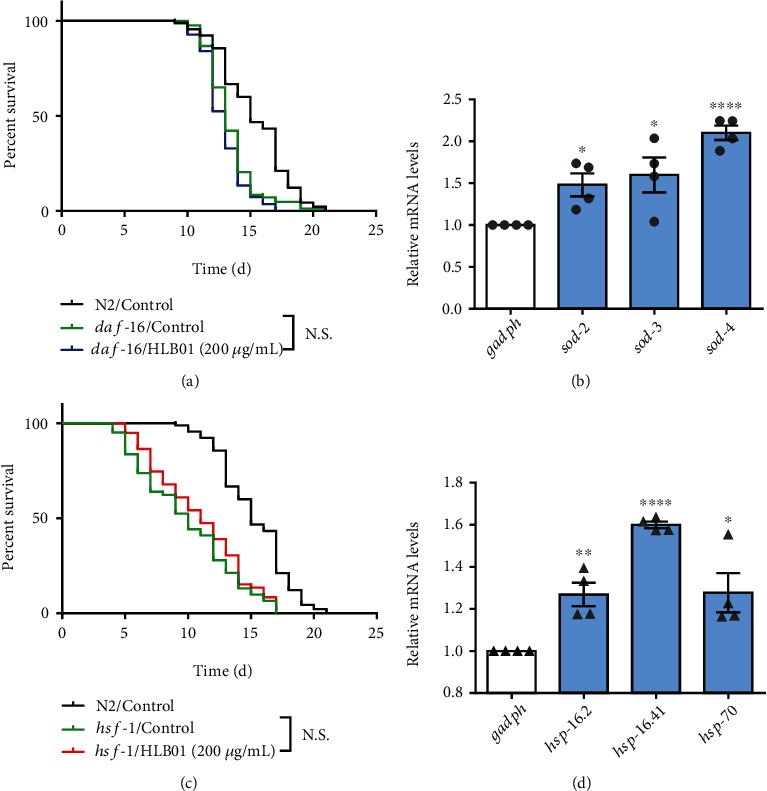
HLB01 enhances stress resistance of *C. elegans* via HSF-1 and DAF-16. (a) HLB01 did not extend the lifespan of *daf-16* mutant worms. (b) HLB01 upregulated the DAF-16-targeted genes (*sod-2*, *sod-3*, and *sod-4*). (c) HLB01 did not extend the lifespan of *hsf-1* mutant worms. (d) HLB01 upregulated the heat shock protein-related genes (*hsp-16.2*, *hsp-16.41*, and *hsp-70*). The log-rank (Mantel-Cox) test was used to calculate the *P* values in (a) and (c). An unpaired *t*-test was used to calculate the *P* values, and error bars represented SEM in (b) and (d). ^∗^*P* < 0.05; ^∗∗^*P* < 0.01; ^∗∗∗∗^*P* < 0.0001; N.S., not significant.

Since HLB01 can extend the lifespan of *C. elegans* and decrease the ROS accumulation during aging, the related longevity signal pathway was explored. We found that HLB01 failed to extend the lifespan in the *daf-16* mutant (Figure 7(a)). Additionally, we also found that HLB01 increased the mRNA levels of superoxide dismutase (*sod*) genes including *sod-2*, *sod-3*, and *sod-4* (^∗^*P* < 0.05, ^∗^*P* < 0.05, and ^∗∗∗∗^*P* < 0.0001), which are the downstream target genes of DAF-16 and encode antioxidant enzymes (Figure 7(b)). These results suggested that HLB01 increases the lifespan of *C. elegans* via activation of DAF-16 and its downstream antioxidant genes.

HSF-1 mainly regulates the expression of heat shock protein (*hsp*) genes to resist the heat stress. We found that HLB01 treatment no longer extended the lifespan in the *hsf-1* mutant (Figure 7(c)). In the HLB01-treated group, *hsp* genes including *hsp-16.2*, *hsp-16.41*, and *hsp-70*, were upregulated (^∗∗∗^*P* < 0.001, ^∗∗∗∗^*P* < 0.0001, and ^∗^*P* < 0.05) (Figure 7(d)). Together, these results suggested that HLB01 extends the lifespan of *C. elegans* related to HSF-1 and increases the heat stress resistance.

## 4. Discussion

The root of *Vicatia thibetica de Boiss* has medicinal and edible value in northwestern Yunnan Province for many years [35, 36]. Homology of medicine and food makes it a potential herbal medicine with high safety. Here, we analyzed the effect of HLB01 on the lifespan and the healthspan including pharyngeal pump and swing of *C. elegans*. As per our knowledge, this is the first report investigating the lifespan-extension and health-improving effect of HLB01 (Figure 3). Not limited to worms, we evaluated antiaging effects in Doxo-treated mice. As the most efficient anthracycline antibiotics used in chemotherapy, Doxo can destroy the cancer cells by blocking the action of the enzyme topoisomerase-II, which prevents the repair of DNA molecules to damage the cell [37]. The senescence-activating effect of Doxo may involve the shortening of telomere length, inhibition of topoisomerases, inducing nucleosome instability, and damaging to DNA [38, 39]. Many works reported that Doxo-induced senescence showed an upregulation of P16^ink4a^, IL1*α*, IL6, FOXO4 foci, and AST [28]. Doxo also induced the upregulation of the *p21* mRNA level in the liver, which is a cyclin-dependent kinase inhibitor and consistently upregulated in response to different aging stimuli [40]. Here, we reported HLB01 could neutralize the Doxo-induced upregulation of AST, ALT, *p21* mRNA, and *γ*H2AX protein levels (Figure 4). *γ*H2AX accumulation is a mark of double-strand DNA breakage which subsequently stimulates the expression of the cyclin-dependent kinase inhibitor P21, an essential mediator of senescence-associated cell cycle arrest [41, 42]. Our results suggested HLB01 counteracted nuclear DNA damage by activating the DNA damage repair pathway, which subsequently alleviates cell cycle arrest and liver damage in Doxo-induced premature aging. Pharmacological evaluation in senescent mice makes HLB01 an antiaging candidate for human health.

Next, in order to further explore the mechanism of the antiaging effect induced by HLB01, transcriptome sequencing was conducted. It was found that HLB01 significantly upregulated the transcription of collagen genes and promoted collagen levels in *C. elegans*. Besides, HLB01 also promoted the expression of collagen type I in human fibroblast (CCC-ESF-1) (Figure 5). It is well-known that collagens are the most abundant and significant ECM proteins in the organism and tissues [14]. A previous study has showed that enhancement of collagen production and ECM remodeling are essential signatures for longevity in worms [11]. The decline in collagen production during aging was observed across the species [30]. For instance, senescent human hepatic stellate cells (HSCs) have reduced the expression of collagen mRNA and protein [43, 44]. Since the enhancement of collagen production can delay aging and promote longevity [11], the increased expression of collagen induced by HLB01 might be one of the essential factors which was responsible for the longevity-promoting property of HLB01. In addition, our findings also suggested that HLB01 could be developed as a natural substance which could promote the collagen and ECM function systemically to exert its antiaging effects.

At present, researches have showed that excess ROS in the organism could cause lipid peroxidation, DNA damage, and changes of proteins, and the oxidative damage caused by ROS was the main factor leading to the aging of the organism [45]. In order to reduce oxidative damage, several studies have been conducted to develop antioxidants [46, 47]. The roots of *Vicatia thibetica de Boiss* have diverse components including umbelliferone, bergapten, ferulic acid, apigenin, *β*-sitosterol, daucosterol, and flavonoid [35, 48], which led us to test the antioxidative ability of HLB01. We found that HLB01 has the free radical scavenging and ROS reducing ability (Figure 6). It means that the antiaging effect of HLB01 is related to the antioxidant pathway to some extent. It is well known that DAF-16 is a transcription factor known to regulate the oxidative stress tolerance and extend the lifespan in *C. elegans* [49]. Many DAF-16 target genes encoding proteins have been predicted to protect the cells from oxidative stress [50, 51], and they are related to HSF-1 which has been known to activate the expression of stress resistance genes for promoting longevity in *C. elegans* [52]. We explored the signal transduction mechanism related to stress resistance. The results showed that the lifespan extension in *C. elegans* induced by HLB01 is DAF-16 and HSF-1 dependent. HLB01 upregulated the downstream genes of DAF-16 and HSF-1 to enhance the oxidative stress resistance and heat stress resistance (Figure 7). Although insulin/insulin-like growth factor-1 signaling implicates collagen remodeling in longevity in *C. elegans* [11], the relationship between DAF-16 and collagen is unknown. Therefore, whether HLB01 promotes the expression of collagen dependent on DAF-16 needs to be further explored.

## 5. Conclusion

After lifespan screening of 836 extracts from Chinese herbal medicine in *C. elegans*, HLB01 was selected for antiaging studies. We for the first time discovered that HLB01 extended the lifespan and healthspan of *C. elegans* as well as neutralized the Doxo-induced premature aging in adult mice. Antiaging effects of HLB01 were mediated via upregulated collagen, antioxidant ability, and stress resistance, indicating HLB01 is beneficial for aging and aging-related disease and could be further developed as a potential Chinese herbal medicine for antiaging in the future. However, the interrelationship in collagen upregulation, antioxidant ability, and stress resistance is unclear, and how these three antiaging mechanisms work in Doxo-induced premature aging mice needs further exploration.

## Data Availability

The data used and analyzed in this study are available from the corresponding author on reasonable request.

## Supplementary Materials

Supplementary Materials Figure S1: cell viability of MRC-5 cells treated with HLB01. Table S1: lifespan data of *C. elegans*. Table S2: primers for specific genes.
