# Supplementary material for: Antiaging Effects of Vicatia thibetica de Boiss Root Extract on Caenorhabditis elegans and Doxorubicin-Induced Premature Aging in Adult Mice
Source: Oxid Med Cell Longev. 2021 Aug 6;2021:9942090. doi: 10.1155/2021/9942090 (PMC8369193; doi:10.1155/2021/9942090)
Supplement: Supplementary Materials — Figure S1: cell viability of MRC-5 cells treated with HLB01. Table S1: lifespan data of C. elegans. Table S2: primers for specific genes. [file 9942090.f1.doc]

**Oxidative Medicine and Cellular Longevity**

**Anti-aging effects of *Vicatia thibetica de Boiss* root extract on *Caenorhabditis elegans* and doxorubicin-induced premature aging in adult mice**

Wenwen Liu,^1^ Yunhui Guan,^2^ Sicong, Qiao,^1^ Jiqun Wang,^1^ Keting Bao,^1^ Zhifan Mao,^1^ Liang Liao,^1^ Alexey Moskalev,^3^ Bei Jiang,^4^ Jin Zhu,^1^ Conglong Xia,^2^ Jian Li,^1, 2, 5^ Zelan Hu.^1^

^1^ State Key Laboratory of Bioreactor Engineering, Shanghai Key Laboratory of New Drug Design, East China University of Science and Technology, 130 Mei Long Road, Shanghai 200237, China

^2^ College of Pharmacy and Chemistry, Dali University, 5 Xue Ren Road, Dali (Yunnan) 671000, China

^3^ Laboratory of Geroprotective and Radioprotective Technologies, Institute of Biology, Komi Science Centre, Ural Branch, Russian Academy of Sciences, 28 Kommunisticheskaya st., Syktyvkar 167982, Russia

^4^ Institute of Materia Medica, Dali University, 5 Xue Ren Road, Dali (Yunnan) 671000, China

^5^ Clinical Medicine Scientific and Technical Innovation Center, Shanghai Tenth People's Hospital, Tongji University School of Medicine, Shanghai 200092, China

Wenwen Liu and Yunhui Guan contributed equally to this work.

Correspondence should be addressed to Zelan Hu; huzelan@ecust.edu.cn. Jian Li; jianli@ecust.edu.cn. Conglong Xia; long7484@126.com.

Copyright © 2021 Wenwen Liu et al. This is an open access article distributed under the Creative Commons Attribution License, which permits unrestricted use, distribution, and reproduction in any medium, provided the original work is properly cited.

**Supplementary materials**

Figure S1. Cell viability of MRC-5 cells treated with HLB01. HLB01 has no cytotoxicity on MRC-5 cells even at a high concentration of 200 μg/mL. An unpaired t-test was used to calculate the *P*-values and error bars represent SEM.

Table S1. Lifespan data of *C. elegans*.

| **Treatment** | **Genotype** | **Mean lifespan（days）** | **Number of worms** | ***P*-Value** |
| --- | --- | --- | --- | --- |
| — | Wild type | 13.19 | 99 | — |
| HLB01 (200 μg/mL) | Wild type | 15.07 | 112 | < 0.0001 |
| HLB01 (400 μg/mL) | Wild type | 14.39 | 111 | < 0.05 |
| — | Wild type | 15.29 | 90 | — |
| — | *daf-16*(mu86) I | 13.39 | 83 | < 0.0001 |
| HLB01 (200 μg/mL) | *daf-16*(mu86) I | 12.85 | 82 | N.S. |
| — | *hsf-1*(sy441) I | 9.97 | 61 | < 0.0001 |
| HLB01 (200 μg/mL) | *hsf-1*(sy441) I | 10.94 | 59 | N.S. |

Table S2. Primers for specific genes.

| **Genes** | **Species** | **Forward Primer (5’ to 3’)** | **Reverse Primer (5’ to 3’)** |
| --- | --- | --- | --- |
| *GADPH* | mouse | GTGGCAAAGTGGAGATTGTTG | AGTCTTCTGGGTGGCAGTGAT |
| *p21* |  | AGCAAAGTGTGCCGTTGTCT | AGAAATCTGTCAGGCTGGTC |
| *GADPH* | *C. elegans* | GGAACTGTTACCTACGATGGAGA | AAACTCCAGTAGACTCGACAACG |
| *col-12* |  | GACAAGATGGAGAGAGTGGATC | TTGAAGCGCTTAATATCCTGGA |
| *col-41* |  | AGATTTGTGTACCCAGGAATGT | GAATATCGTGAGTGGTTGCAAG |
| *col-63* |  | CCAGAAGGAGATGAAGGAACTC | TGTCCATCTTGTCCATTCTCTC |
| *col-77* |  | TGTACAACTACATGCAGACTGT | TCTTAACTCCCTTGATACGCTC |
| *col-138* |  | CGATTTCTGCTTTGAGTGTGAG | GCTTCTCCCTTTGGTCCTT |
| *col-175* |  | TCGCTATGCTCTGTTCTTGTTA | GATTCACATTGGCAGCTATCAG |
| *sod-2* |  | GATACTGTCCAAAGGGAAAGAT | GTAGTAAGCGTGCTCCCAGA |
| *sod-3* |  | ATCTACTGCTCGCACTGCTT | TTTCATGGCTGATTACAGGTT |
| *sod-4* |  | GCACCAGATGACTCGAACA | GTCCACTTAATGAGGCAAGA |
| *hsp-16.2* |  | GTACGCTATCAATCCAAGGAGA | GGAATTGATCTTCCTTGAACCG |
| *hsp-16.41* |  | AAGTTTTCGGTTCAACTCGATG | TCCCTTCAATTTTGAGCTCTCT |
| *hsp-70* |  | GATCGAATTAGCTCGCGTAATC | TCCGCTAATGTATTACGTTCCA |
